# Glutamine’s double-edged sword: fueling tumor growth and offering therapeutic hope

**DOI:** 10.3389/fimmu.2025.1578940

**Published:** 2025-04-10

**Authors:** Liguang Fang, Dandan Gao, Zuomin Jiang, Guoliang Li, Ming Li

**Affiliations:** ^1^ College of Traditional Chinese Medicine, Shandong University of Traditional Chinese Medicine, Jinan, Shandong, China; ^2^ The First Clinical Medical College, Shandong University of Traditional Chinese Medicine, Jinan, Shandong, China; ^3^ Jinan Zhangqiu District Hospital of Traditional Chinese medicine, Jinan, Shandong, China; ^4^ Jinan Nanshan People's Hospital, Jinan, Shandong, China; ^5^ Shandong University of Traditional Chinese Medicine College of Ophthalmology and Optometry, Jinan, Shandong, China

**Keywords:** combination therapy, glutamine metabolism, immune microenvironment, metabolic reprogramming, tumors

## Abstract

Tumor metabolic reprogramming is a highly complex process that enables tumor survival in the presence of limited nutrients, involving multiple signaling pathways, non-coding RNAs (ncRNAs), and transcription factors. Lately, glutamine has been found to enhance the growth, spread, and drug resistance of cancer cells, while also fostering an immunosuppressive microenvironment that aids tumor development. However, in some tumors, such as pancreatic cancer and melanoma, additional glutamine can inhibit the proliferation of tumor cells, and this mechanism is closely related to the regulation of the immune microenvironment. Therefore, further exploration of glutamine metabolism in tumors is essential for understanding the pathogenesis of cancer and for developing new metabolically targeted therapies. We systematically review the latest research on the reprogramming of glutamine metabolism and its role of tumor growth, spread, and immune system regulation. Additionally, we review the clinical research progress on targeted glutamine therapies and their application in combination with current anti-tumor treatments. Ultimately, we address the challenges and prospects involved in resistance to anti-cancer strategies aimed at glutamine metabolism.

## Graphical Abstract

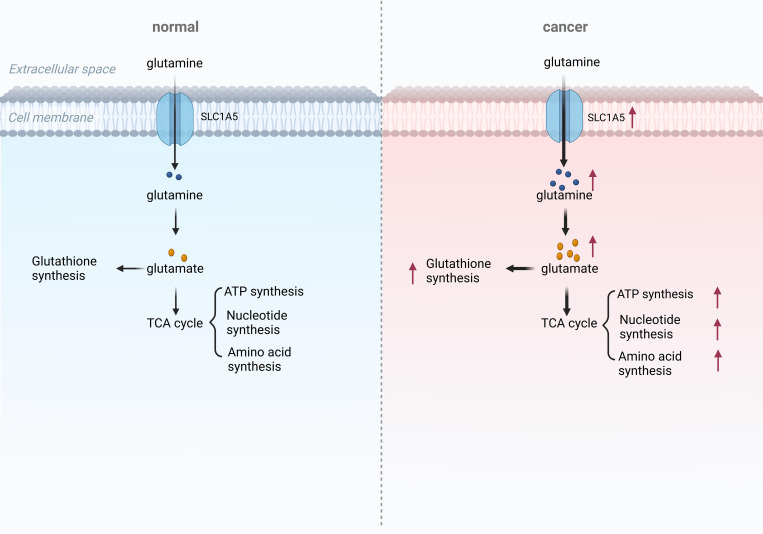

## 1 Background

Metabolic reprogramming is a hallmark of malignant tumors (1). Cancer cells sustain their uncontrolled proliferation under conditions of hypoxia and nutrient deficiency by modifying their synthesis and catabolism (2). Consequently, a variety of anti-metabolic chemotherapy agents, including methotrexate and 5-fluorouracil, are widely utilized in clinical practice (3, 4). However, while these anti-metabolic drugs effectively target cancer cells, they also inflict damage on normal cells, including immune cells. Furthermore, side effects such as dose limitations and cardiotoxicity have significantly restricted their clinical application (5). Therefore, developing metabolic-targeted therapies with tumor selectivity is of great importance for enhancing the efficacy of cancer treatment in clinical settings.

Glutamine (Gln) metabolic reprogramming is a crucial aspect of tumor metabolic reprogramming and plays a significant role in the initiation and progression of tumors. Targeting tumor Gln metabolism has emerged as a prominent focus in tumor metabolic therapy. Gln, a nonessential amino acid abundant in the bloodstream, along with its metabolites lutamate and αketoglutarate (α-KG), serves as a vital source of carbon and nitrogen, providing essential raw materials for the synthesis of nucleotides and amino acids (6, 7). According to recent studies, various tumor cells enhance their Gln usage by increasing the expression of Gln transporters and enzymes involved in metabolism. This regulation affects the balance of reactive oxygen species (ROS), as well as the sensitivity of tumor cells to radiotherapy and chemotherapy, and their immune response. “Gln addiction” has emerged as a hallmark of many cancers (8, 9). Consequently, numerous studies have sought to inhibit tumor progression through the targeted inhibition of Gln metabolism. Presently, the primary metabolic inhibitors of Gln include those that inhibit Gln uptake, glutaminase (GLS) inhibitors, and Gln antimetabolites. Notably, some of these inhibitors (CB-839, DRP-104) have progressed to phase I-II clinical trials for cancer treatment (10, 11).

Although some Gln metabolic inhibitors have entered clinical trials, the heterogeneity among tumors complicates their effectiveness. Different tumors utilize Gln in distinct ways, which means that Gln inhibitors may have varying roles across different tumor types. Moreover, Gln is an essential substrate for the immune system, and prolonged use of Gln metabolic inhibitors might weaken the anti-tumor immune response (9, 12). Thus, it is essential to investigate how Gln metabolism affects tumor cell proliferation and immune cell function to aid in creating novel metabolic therapies. This paper summarizes the successful research on Gln metabolic reprogramming in tumors and reviews the clinical progress of anti-tumor therapies targeting Gln metabolism, aiming to provide insights for the development of innovative metabolic-targeted treatments.

## 2 The role of Gln metabolism in tumor

**Figure 1:**
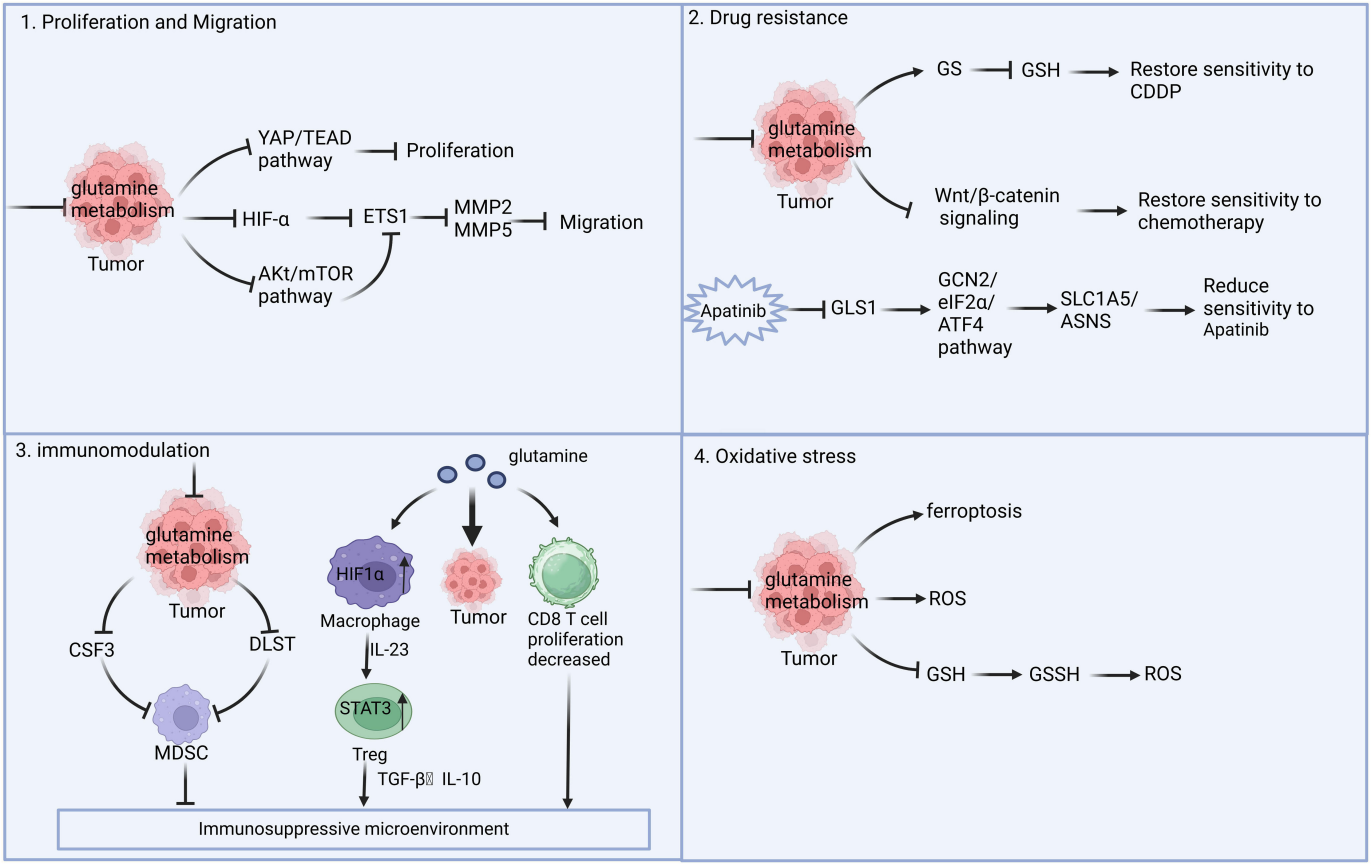
Role of Gln metabolism in tumor. Gln metabolism can promote tumor proliferation and metastasis through the HIF-α and Akt/mTOR signaling pathways, and it can induce drug resistance via the Wnt/β-catenin pathway. Additionally, Gln metabolism can contribute to the formation of an immunosuppressive microenvironment and oxidative stress, thereby promoting tumor progression. Inhibiting Gln metabolism may serve as an effective anti-tumor strategy.

Emerging evidence shows that Gln metabolism is involved in tumor progression in two specific ways (13). On one hand, Gln can facilitate the malignant progression of tumors by reducing sensitivity to radiotherapy and chemotherapy, as well as by regulating the formation of the immune microenvironment and maintaining redox homeostasis (**Figure 1**). On the other hand, nutritional stress resulting from Gln deprivation can enhance the activity of anti-tumor immune cells and inhibit tumor progression. Therefore, understanding the role of Gln metabolism in tumor progression is crucial.

### 2.1 The impact of Gln metabolism on tumor promotion

#### 2.1.1 Promotion of tumor cell proliferation and metastasis

Gln metabolism is intricately linked to tumor proliferation and migration. Due to the rapid proliferation and metabolism of tumor cells, glycolysis is highly active, and the tumor microenvironment (TME) often exhibits characteristics of hypoxia and glucose deprivation. In a hypoxic and glucose-deprived TME, The metabolism of Gln is essential for tumor cells to survive and multiply (14). Gln plays a vital role in maintaining cancer cell viability, acting as a substrate for numerous metabolic activities, such as the Krebs cycle, maintaining redox balance, and synthesizing vital cellular components like nucleic acids, fatty acids, glutathione (GSH), and other amino acids (15). Triple-negative breast cancer (TNBC) and KRAS-mutant pancreatic cancer often exhibit significant Gln addiction, with survival rates positively correlated to Gln metabolic activity. In TNBC, both ASCT2 and LAT1 are overexpressed (16). The high expression of ASCT2 enhances Gln uptake and metabolism, which activates the mTORC1 nutrition-sensing pathway (16).

Additionally, metabolomic analyses reveal low levels of Gln and elevated levels of glutamate in TNBC, suggesting that TNBC is more dependent on Gln and more sensitive to therapies targeting Gln breakdown compared to other breast cancer subtypes (17). Pancreatic cancer relies on glucose and Gln metabolism, with clearance pathways primarily mediated by the Ras pathway to fulfill its energy requirements. Carcinogenic RAS-driven signals promote Gln metabolism, significantly contributing to the development, survival, and invasiveness of pancreatic tumor cells. This results in the phenomenon of Gln metabolism addiction in pancreatic tumor cells (18).

The role of Gln is critical in both the proliferation and metastasis of cancer cells. When Gln addiction is disrupted due to Gln deprivation, the invasive and metastatic capabilities of tumor cells are significantly diminished. Adhikary et al. demonstrated that reducing Gln levels, inhibiting the transmembrane transport of Gln (using V-9302), or blocking Gln metabolism (with CB-839) markedly decreased mesothelioma cell proliferation, spheroid formation, invasion, and migration. Mechanistic studies have revealed that the application of Gln metabolic inhibitors or the knockdown of the transmembrane protein SLC1A5 can inhibit the YAP1/TEAD signaling cascade, which is known to promote mesothelioma invasion and metastasis (19). Furthermore, numerous studies have demonstrated that leukemia cells exhibit a significant dependence on Gln. Yucel et al. observed that leukemia cell lines, such as K562, NB-4, and HL-60, showed reduced cell proliferation and increased expression of Gln synthetase (GS) protein under conditions of Gln deficiency (20). Later research showed that GS protein can support tumor cell growth even when Gln is scarce and was closely associated with the Gln addiction of leukemia cell lines. Additionally, ETS1, a key regulator of epithelial-mesenchymal transition (EMT) and metastasis in ovarian cancer cells, is also modulated by Gln. Prasad et al. found that Gln deprivation inhibited the expression of ETS1 in PA1 cells in a HIF1-dependent manner. Moreover, Gln deprivation reduced the production of matrix metalloproteinases (MMP2 and MMP9) in SKOV3 cells by inhibiting the nuclear translocation of ETS1, thereby diminishing the migratory and invasive capabilities of ovarian cancer cells (21).

#### 2.1.2 Promoting drug resistance in tumor cells

The dysregulation of Gln metabolism is closely associated with the development of tumor resistance (22, 23). Studies have demonstrated that in pancreatic cancer, targeting Gln metabolism in conjunction with ERK signaling pathway inhibitors can halt pancreatic cancer cell metabolism and treatment resistance, thereby enhancing treatment outcomes (24). Like other malignancies, nonsmall cell lung cancer (NSCLC) requires Gln for proliferation. Studies have shown that drug resistant tumor cells are highly dependent on Gln utilization, with glutamate dehydrogenase 1 (GLUD1) serving as a key factor in Gln addiction in acquired drug-resistant NSCLC cells. GLUD1mediated production of alpha-ketoglutarate (α-KG) and the accumulation of ROS trigger migration and invasion by inducing the expression of Snail (25). Broad-spectrum Gln metabolic antagonists can inhibit the uptake and utilization of Gln by tumor cells, thereby suppressing tumor cell proliferation and increasing sensitivity to epidermal growth factor receptor tyrosine kinase inhibitors (EGFR-TKIs) in NSCLC (26). Ocitinib-resistant cells can enhance glucose and Gln uptake, while inhibiting Gln uptake and metabolism in NSCLC, effectively reducing the proliferation of ocitinib resistant cells and promoting cell apoptosis (27). Given the Gln dependence of ocitinib-resistant lung cancer cells, targeting GLS1 alone has limited anticancer effects. Clinical trials have demonstrated that a dual-targeting approach, which simultaneously inhibits ASCT2 and GLS1, may be a promising strategy for sensitizing ocitinib-resistant cells (28). The application of broad spectrum Gln metabolic antagonists can mitigate drug resistance in NSCLC and improve the clinical efficacy of osimertinib (29).

In ovarian cancer, the levels of Gln, glutamate, and GSH in the cisplatin-resistant cell line A2780cis were significantly higher than those in the A2780 cells, while the expression of GS decreased. Treatment of A2780cis cells with 5-aza-2’-deoxycytidine, a DNA demethylation agent, restored GS expression and reduced cisplatin resistance. Gln deprivation can enhance the sensitivity of A2780cis cells to cisplatin by promoting the methylation of the GS promoter, inhibiting GS synthesis, and downregulating intracellular GSH levels (30). Myc can induce the upregulation of GS by enhancing the transcriptional activity of its target, thymine DNA glycosylase (TDG). TDG facilitates the active demethylation of the GS promoter, leading to increased expression (31). In liver cancer, caraglipzin (CANA) can reduce aerobic glycolysis in HCC by targeting pyruvate kinase M2 (PKM2). In the cytoplasm, PKM2 can directly associate with the transcription factor c-Myc to create a complex, enhancing the phosphorylation of c-Myc at Thr58 and facilitating its ubiquitination and degradation. The reduction of c-Myc decreases the expression of GLS1, a key enzyme in Gln metabolism, resulting in impaired Gln utilization and increased sensitivity to chemotherapy in liver cancer (32). Apatinib, which is a tyrosine kinase inhibitor, is used to treat different types of cancer. However, the emergence of apatinib resistance has significantly limited its clinical application. One study demonstrated that apatinib inhibited the expression of GLS1, activated the GCN2/eIF2α/ATF4 pathway, and promoted the expression of SLC1A5, thereby reprogramming Gln metabolism in NSCLC and weakening the response of tumor cells to apatinib (33).

Furthermore, Gln metabolism can also exert epigenetic influences on tumor resistance. Gln metabolism directly or indirectly influences epigenetic processes, such as DNA methylation and histone modification, through mechanisms of “substrate supply” and “cofactor regulation. Gln is metabolized by GLS to produce alpha-ketoglutaric acid (α-KG), which serves as a crucial cofactor for TET enzymes that catalyze DNA demethylation, as well as for histone demethylases like JHDM. In pancreatic cancer, Gln confers resistance to tumor necrosis factor-associated apoptosis-inducing ligand (TRAIL) in pancreatic ductal adenocarcinoma (PDAC) cells through KDM4C-mediated epigenetic regulation of cFLIP. Inhibition of Gln metabolism significantly reduces cFLIP levels, leading to the formation of the death-inducing signaling complex mediated by TRAIL. Supplementation with α-KG effectively reverses the decrease in cFLIP levels caused by the inhibition of Gln breakdown and enhances resistance to TRAIL (34). In KRAS-mutated colon cancer cells, SLC25A22-mediated Gln breakdown reduces H3K4me3 methylation, stimulates the activation of the Wnt/beta-catenin signaling pathway, and diminishes the sensitivity of colon cancer cells to chemotherapeutic agents (35). Therefore, targeting Gln metabolism may represent a novel therapeutic strategy against tumor resistance.

#### 2.1.3 Induction of an immunosuppressive microenvironment

Gln is a conditionally essential amino acid crucial for the metabolism of both tumor and immune cells. Activated T cells that upregulate Gln transporters also exhibit signs of GLS-dependent activity (36). In inflammatory diseases, T cells that are deficient in GLS are not fully activated and quickly become depleted (36). In models of viral infection, the expansion of GLS-deficient CD8+ T cells was significantly reduced, further underscoring the importance of GLS activity in CD8+ T cell function. In malignant tumors, IFNγ produced by CD8+ T cells increases with rising Gln concentrations, indicating that immunotherapy targeting tumors also relies on Gln-dependent activation of CD8+ T cells (12). Thus, not only do tumor cells exhibit Gln-dependent characteristics, but CD8+ T cells do as well. In TME, tumor cells compete with various immune cells for the utilization of Gln. Tumor cells activate multiple carcinogenic pathways, resulting in the increased expression of Gln transporters and enzymes related to metabolism. This activation promotes the uptake of Gln by tumor cells, leading to a significant consumption of Gln within the TME (14). Unfortunately, immune cells struggle to match the uptake and metabolic capacity of tumor cells (15). At the genetic level, it has been observed that the expression of Gln metabolism-related genes in tumor cells is significantly higher than that in CD8+ T cells, underscoring the metabolic advantage held by tumor cells (16). Immune cells require a specific amount of Gln, and a reduction in Gln levels can compromise their integrity and functionality (16). The predatory consumption of Gln by tumor cells limits the functionality of CD8+ T cells, ultimately leading to immune escape.

In the microenvironment of liver cancer tumors, Gln deficiency promotes the invasion of myeloid cells expressing the immunosuppressive receptor GPR109A, facilitating immune evasion.

Inhibition of GPR109A can be accomplished through the Gln metabolism/ER stress/GPR109A axis.

This intervention results in a reduced abundance of granulocytic myeloid-derived suppressor cells (G-MDSCs) and M2-like tumor-associated macrophages (TAMs), thereby triggering an anti-tumor response from CD8+ T cells (37). In ovarian cancer, the metabolic inhibitors 6-diazo-5-oxo-Lleucine (DON) and calcium carbonate were encapsulated using folate-targeted nanoparticles (FADCNPs). FA-DCNPs inhibit glutamate production through folate receptors (FOLR). Targeting M2phenotypic TAMs reduces the polarization of macrophages toward the M2 phenotype and raises the amount of M1-TAMs, which in turn strengthens the anti-tumor immune microenvironment (38). In prostate and bladder cancer, the Gln metabolism antagonist JHU083 inhibits TAM Gln metabolism, resulting in increased glycolysis, disruption of the tricarboxylic acid (TCA) cycle, and impairment of purine metabolism. This promotes stem-like phenotypes in CD8+ T cells and diminishes the population of regulatory T cells (Treg), thereby enhancing the anti-tumor immune response (39).Chen et al. found that the distribution of Gln between immune cells and tumor cells may reshape the tumor immune microenvironment. When the ability of tumor cells to metabolize Gln is significantly greater than that of CD8+ T cells, the tumor-killing ability of the CD8+ T cell subgroup is inhibited. Following treatment with the Gln metabolism inhibitor JHU083, tumor cell growth was suppressed, and the number of CD8+ T cells increased, thereby enhancing the efficacy of the PD-1 blocker (40).

At the same time, Fu et al. found in their study that in clear cell renal cell carcinoma (ccRCC) tumor cells, endogenous Gln metabolism might reduce the anti-tumor effectiveness of T cells by promoting the Treg cell response (41). According to the proposed mechanism, Gln consumption by ccRCC tumor cells leads to local depletion of extracellular matrix Gln, thereby activating the HIF1α/IL-23 signaling pathway in tumor-infiltrating macrophages. This activation promotes the secretion of interleukin-10 (IL-10) and transforming growth factor beta (TGF-β) by Treg, ultimately inhibiting the anti-tumor activity of T cells. In addition, medullary-derived suppressor cells (MDSCs) play a crucial role in the formation of an immunosuppressive TME. Oh et al. found that blocking Gln metabolism can enhance tumor-specific immunity by inhibiting the transcription of CSF3 in tumor cells, which decreases the generation and attraction of MDSCs and stimulates the creation of pro-inflammatory TAMs (42). Furthermore, Gln deprivation inhibits the formation of MDSCs by downregulating the expression of dihydrolipoamide succinyl transferase (DLST) in myeloid cells. Therefore, targeting Gln metabolism within the TME may inhibit the development of an immunosuppressive environment and improve the efficacy of immunotherapy.

#### 2.1.4 Regulation of tumor cell redox homeostasis

Gln regulates the redox status of cancer cells by influencing the production of mitochondrial ROS. Gln is transported to cells where it acts as a starting material for the production of different amino acids, proteins, nucleotides, and other important biological molecules. It also provides reduced coenzyme II (NADPH) and GSH to maintain redox balance. GSH is a crucial intracellular small-molecule reducing agent that effectively removes ROS and plays a vital role in the redox homeostasis of tumor cells (43). Consequently, the inhibition of Gln metabolism is associated with elevated ROS levels, which in turn promotes apoptosis in tumor cells (44). In studies of human glioblastoma, the absence of exogenous Gln significantly reduces glutamate levels, resulting in the conversion of the dimer GLS1 into a self-assembled, extremely low Km filamentous polymer that further depletes intracellular Gln. This scarcity of intracellular Gln hampers the synthesis of asparagine and proteins encoded by the mitochondrial genome, ultimately leading to ROS-induced apoptosis in tumor cells (45). In lung cancer, spermidine/spermidine N1-acetyltransferase 1 (SAT1) serves as a rate-limiting enzyme in polyamine catabolism. Its activation enhances the conversion of Gln to glutamate and promotes subsequent GSH synthesis, while also leading to ROS accumulation, which inhibits the proliferation of lung cancer cells and promotes apoptosis (46).

Gln regulates the redox state of cancer cells and influences mitochondrial ROS production. Furthermore, oxidative stress can exacerbate tumor cell damage and enhance radiosensitivity by inducing ferroptosis. Through targeted metabolomics analysis, Yuan et al. discovered that the amino acid content in radiation-resistant HepG2 cells significantly increased under iron death stress. Nacetyl Gln, a derivative of Gln, is closely associated with the redox homeostasis of tumor cells. Further studies revealed that the deprivation of Gln induced iron death in liver tumor cells and heightened their radiosensitivity, while Gln supplementation completely reversed the effects of iron death. Therefore, Gln deprivation-induced iron death in tumor cells may represent a novel therapeutic strategy for liver tumors (47). Research has shown that miR-137 inhibits ferroptosis by directly interacting with the Gln transporter SLC1A5 in melanoma cells. Overexpression of miR137 suppresses SLC1A5, resulting in decreased glutamine uptake and higher levels of malondialdehyde (MDA). Reducing miR-137 boosts Erastin’s antitumor effects by encouraging ferroptosis in both laboratory and live models (48). Extended limitation of Gln or administration of the Gln antagonist 6-diazo-5-oxo-L-norleucine (DON) leads to growth suppression and triggers the ferroptosis pathway in PDAC. The epigenetic regulator Paxip1 enhances the increase of H3K4me3 and transcription of Hmox1 following DON treatment. Additionally, it increases the levels of ferroptosis markers (e.g., Slc7a11 and Gpx4), which predispose PDAC cells to ferroptosis under conditions of Gln deprivation, thereby providing a combined strategy for PDAC therapy (49). Additionally, Rashmi et al. found that Gln deprivation resulted in a decrease in total reduced GSH and an increase in GSH disulfide levels in cervical cancer cells, thereby elevating intracellular oxidative stress and inducing apoptosis (50).

Inhibiting Gln metabolism may serve as an effective anti-tumor strategy.

### 2.2 Anti-tumor effects of Gln metabolism

Gln, a crucial nutrient in the TME, supports the growth of tumor cells. Consequently, the inhibition of Gln leads to a deficiency of intracellular nutrients, which can impede tumor progression. However, other studies have indicated that nutritional stress resulting from Gln deprivation may promote tumor development. For instance, Recouvreux et al. discovered that nutritional stress due to Gln deprivation enhances the metastasis of PDAC. Mechanistically, Gln deprivation increases slug expression by triggering the ATF4 and MEK/ERK signaling pathways, thereby inducing EMT and promoting the metastasis of PDAC cells. It has been found that cancer-associated fibroblasts (CAFs) have a greater dependence on Gln than tumor cells do and exhibit greater sensitivity to GLS inhibition (51). Mechanistically, CAFs can migrate along the Gln concentration gradient toward nutrient-rich neighboring tissues, thereby facilitating the invasion of tumor cells into surrounding areas. Exogenous Gln supplementation has been shown to inhibit tumor growth and enhance the immune response against tumors (52, 53). In melanoma, Gabra et al. conducted a metabolomic analysis revealing that dietary Gln supplements increase the concentration of α-KG within tumor cells and reduce the methylation of H3K4me3 (54). This reduction inhibits the epigenetically activated carcinogenic pathway, thereby suppressing tumor growth. Furthermore, Gln deprivation enhances resistance to immunotherapy in lung cancer by promoting autophagy-dependent degradation of the interferon-gamma receptor (IFNGR1) (55).Gabra et al. found that dietary Gln supplementation can elevate Gln levels in the TME, decrease the expression of immunosuppressive genes such as Ctla4 and Nrp1 in Tregs, inhibit Treg differentiation, and consequently enhance the tumor immune response (52).

## 3 Regulation of Gln metabolism in tumors

### 3.1 ncRNAs regulate Gln metabolism

ncRNAs play a crucial role in the occurrence and development of tumors. Numerous studies have demonstrated that ncRNAs regulate Gln metabolism in tumor cells by modulating Gln transporters and metabolic enzymes. For instance, Cai et al. found that circ_0000808 was upregulated in NSCLC cells, where it enhanced the expression of the Gln transporter SLC1A5 by interacting with miR1827, thereby promoting Gln metabolism and facilitating the progression of NSCLC (56). In esophageal cancer, Wu et al. found that circ_0001273, as a cancer-promoting factor that upregulates SLC1A5 expression by binding to miR-622, which increases Gln uptake by tumor cells, consequently enhancing tumor growth and inhibiting cell apoptosis (57). In addition, the SLC38A2 and SLC7A5 transporters play crucial roles in Gln transport within cells. Liu et al. discovered that the CAF-derived exosome LINC01614 can activate the NF-κB signaling pathway by interacting with ANXA2 and p65 (58). This interaction upregulates the expression of SLC38A2 and SLC7A5 in cancer cells, enhances Gln uptake, and stimulates the expansion and migration of tumor cells. Furthermore, ncRNAs can also trigger the transcription of metabolic enzymes in a direct or indirect manner. For instance, Qian et al. found that circ_0001093 can competitively bind to miR-579-3p, leading to the upregulation of GLS, which stimulates Gln metabolism and the malignant progression of esophageal squamous cell carcinoma (ESCC) (59). Kaur et al. (60) utilized next generation sequencing (NGS) and computational analysis to demonstrate that miR-23b-3p, acting as a tumor suppressor, can inhibit tumor Gln metabolism by targeting GLS1, thereby enhancing the sensitivity of liver tumor cells to sorafenib. Glutamic pyruvate aminotransferase 2 (GPT2) is a key enzyme in the Gln decomposition pathway (61). Zhao et al. found that LncRNA UCA1 can specifically bind to and promote the interaction between heteronuclear ribonucleoprotein (hnRNP I/L) and the GPT2 promoter, resulting in increased GPT2 expression, which subsequently enhances the Gln-fueled TCA cycle and tumor development (62).

ncRNAs also influence tumor Gln metabolism by modulating various signaling pathways (**Table 1**). In hepatoblastoma (63), circHMGCS1 upregulates the expression of IGF through competitive binding to miR-503-5p, thereby activating the PI3K-Akt signaling pathway, which promotes Gln decomposition and cell proliferation. Additionally, Cao et al. found that circLMO7 activated the WNT2/β-catenin signaling pathway by releasing miR-30a-3p, which inhibits its target gene WNT2 (64). This process upregulates GLS expression and promotes the proliferation, migration, and Gln metabolism of gastric cancer (GC) cells.

**Table 1:** ncRNA regulates tumor Gln metabolism.

Classification	Name	Expression level	Targets/Mechanisms	References
circRNA	circ_0000808 circ_0001273 circ_0001093	up up	upregulating SLC1A5 expression upregulating GLS expression	(56) (57) (59)
	circHMGCS1	up	activating PI3K-Akt pathway	(63)
	circLMO7	up	activating WNT2/β-Catenin pathway	(64)
lncRNA	LINC01614	up	activating the NF-κB signaling pathway, upregulating SLC38A2 and SLC7A5	(58)
	UCA1	up	upregulating GPT2 expression	(62)
miRNA	miR-23b-3p	down	inhibiting GLS expression	(60)

### 3.2 Oncogenes regulate Gln metabolism

Some oncogenes have the ability to impact tumor development by modulating key transporters and enzymes in Gln metabolism (**Table 2**). CEMIP, identified as an oncogene, enhances the stability and nuclear accumulation of c-Myc by inhibiting the ubiquitination of this proto-oncogene. The SLC1A5 promoter can be directly targeted by c-Myc, causing an upregulation of SLC1A5 expression in cells and facilitating Gln uptake. In oral cancer, c-Myc also promotes cell proliferation by increasing the activity of GLS and GS, thereby improving the Gln metabolism of tumor cells.

**Table 2:** Oncogenes and tumor suppressor genes regulate tumor Gln metabolism.

Classification	Name	Expression level	Targets/Mechanisms	References
oncogene	CEMIP, c-Myc	up	upregulating SLC1A5 expression	(65, 66)
	c-Myc	up	upregulating GLS, GS expression	(67)
Tumor suppressor gene	P53 Rb	down down	inhibiting GLS2 expression inhibiting SLC1A5 expression	(68) (69)

In addition, the absence of the P53 tumor suppressor gene can obstruct Gln utilization by decreasing GLS2 levels inside cells and boosting ROS levels (68). Rb, another tumor suppressor gene, directly inhibits the cell cycle by suppressing the transcription factor E2F family (70). One study found that Rb inhibits the expression of SLC1A5 and the activity of GLS1 by regulating E2F-3, consequently blocking the intake and metabolic processing of Gln.

## 4 Targeting Gln to enhance clinical treatment of tumors

**Figure 2:**
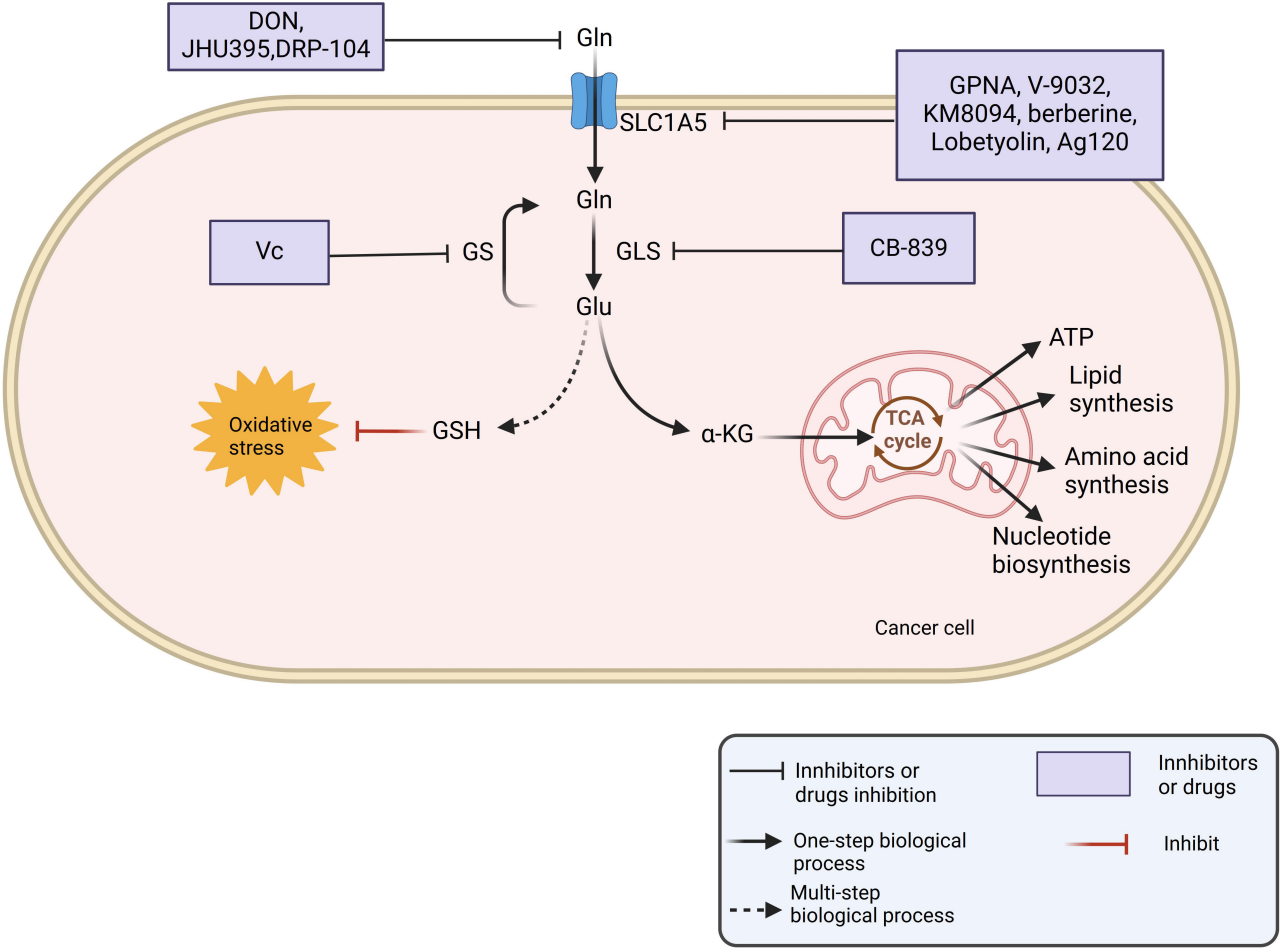
Gln is taken up by the cell through SLC1A5 and is converted to glutamate by the action of GLS. Glutamate is converted to α-KG for the biosynthesis of ATP, nucleic acids, amino acids and lipids. At the same time, glutamic acid and cysteine, glycine synthesize GSH, regulate the redox state of the cell. Drugs such as DON and JHU395 can inhibit the transmembrane transport of Gln by targeting Gln transporters. Additionally, CB839 can disrupt Gln metabolism by inhibiting the enzyme GLS.

The reprogramming of Gln metabolism is strongly linked to the aggressive development of tumors. Consequently, the development of therapeutic agents that disrupt various aspects of Gln metabolism may represent a promising anticancer strategy (**Figure 2**). In this review, we summarize several drugs and inhibitors that specifically target Gln metabolism.

### 4.1 Imaging of Gln metabolism predicts treatment response

Abnormal Gln metabolism has facilitated the use of non-invasive imaging techniques for early tumor diagnosis and treatment monitoring, such as 18F-(2S,4R)-4-fluoroglutamine (18F-FGln) PET and L-(17)-glutamine (11C-Gln) positron emission tomography (PET) (71). Imaging examinations are crucial for the early identification and prognosis evaluation of cancer patients. Compared to traditional imaging methods, PET offers unique advantages in studying cellular metabolism, including higher spatial resolution and sensitivity. 18F-FGln is a Gln analogue that serves as a PET radiotracer for imaging tumor Gln flux and metabolism. For instance, Xu et al. observed significant heterogeneity in 18F-FGln uptake patterns among different lesion types while investigating optimal imaging phases for various cancers. In breast cancer, administering 18F-FGln PET 10 minutes after the injection of 18F-FGln may enhance contrast resolution (SUR) for patients. Therefore, adjusting the timing of PET imaging for different cancer types could further improve diagnostic accuracy and optimize personalized treatment plans (72). In addition, while studying the pharmacokinetic properties of 18F-FGln in cancer patients, Grkovski et al. found that dynamic data analysis significantly enhanced the evaluation of the therapeutic effects of drugs targeting tumor Gln metabolism (73, 74). 11C-Gln is another PET tracer. A clinical study has demonstrated that 11CGln is well tolerated by patients with metastatic colorectal cancer (CRC) and can be visualized noninvasively in multiple organs, including the lungs, brain, and bones (75). Therefore, the use of 18FFGln and 11C-Gln as radioactive tracers in non-invasive imaging through PET may aid in monitoring patients’ treatment responses, improving early diagnosis rates, and facilitating precise cancer treatment.

18F-FGln and 11C-Gln are imaging agents used for assessing Gln metabolism in tumors. However, their synthesis processes are complex. The half-life of 11C-Gln is only 20 minutes, while that of 18F-FGln is approximately 110 minutes. This short half-life significantly impacts the efficiency of labeling after injection into a patient’s body, thereby limiting their clinical applications. In addition to the inherent challenges associated with these agents, Gln metabolism lacks tumor specificity and involves multiple metabolic pathways within cells, which can lead to false positives in areas of high metabolic activity. Furthermore, the high heterogeneity of tumors means that different tumor cells exhibit varying degrees of dependence on Gln, resulting in inconsistent sensitivity to these imaging agents. Due to these limitations, Gln metabolism imaging technology primarily focuses on animal models or small-scale clinical trials, and there is a lack of large-scale population data (72). However, as research into the mechanisms of Gln metabolism in tumor cells advances, the application of Gln metabolism imaging technology is expected to expand. Reason: Improved clarity, vocabulary, and technical accuracy while maintaining the original meaning.

### 4.2 Targeting Gln metabolism to inhibit tumor proliferation

#### 4.2.1 Inhibition of Gln uptake

Tumor survival and proliferation are heavily dependent on Gln, a hydrophilic amino acid that must be transported into tumor cells by transmembrane proteins, such as SLC1A5, SLC6A14, and SLC38A1/2. In the TME, immune cells often prioritize glucose uptake, leading to the upregulation of various Gln transporters in different cancer types, including TNBC, esophageal cancer, stomach cancer, and lung cancer. Consequently, tumor cells exhibit a heightened affinity for Gln absorption within the TME, depleting the limited Gln supply and exerting an immunosuppressive effect (76). In lung adenocarcinoma, CAF-specific long non-coding RNA, LINC01614, packaged in exosomes derived from CAFs, interacts directly with ANXA2 and p65 to promote NF-κB activation. This interaction results in the upregulation of Gln transporters SLC38A2 and SLC7A5, facilitating Gln uptake by lung cancer cells and promoting tumor proliferation (58).

Gln enters cells through transporters such as SLC1A5, SLC38A2, and SLC7A5. Among these, SLC1A5 is a key transporter of Gln and is found at higher levels in various cancers, such as breast cancer, liver, and pancreatic cancer. Its upregulation is significantly associated with poor patient prognosis (77). Gene knockout or pharmacological inhibition of SLC1A5 reduces Gln uptake by cancer cells, thereby impairing their proliferation and survival. Consequently, numerous studies have sought to inhibit tumor progression by identifying specific inhibitors of SLC1A5. One common SLC1A5 inhibitor, GPNA, along with its derivative V-9032, exerts anti-tumor effects by blocking Gln uptake, inhibiting cell proliferation, and disrupting the intracellular redox state. However, both GPNA and V-9032 have notable drawbacks, including poor selectivity, a tendency for off-target effects, and cytotoxicity. Furthermore, some studies have indicated that V-9032 does not directly target SLC1A5 but instead inhibits the anti-tumor effects of SLC38A2 (78). As a result, these compounds have not progressed to clinical trials as targeted inhibitors. Monoclonal antibodies (mAbs), as natural immune molecules, exhibit greater specificity and stability compared to traditional small molecule drugs (79). For instance, in a study conducted before human trials, Sasakawa et al. demonstrated that KM8094, a humanized monoclonal antibody targeting SLC1A5, can inhibit cellular Gln uptake by binding to SLC1A5. This action leads to a reduction in intracellular GSH levels, resulting in increased oxidative stress, which induces apoptosis and cell cycle arrest. Furthermore, KM8094 also suppresses tumor growth through antibody-dependent cellular cytotoxicity (ADCC) (80). Consequently, KM8094 may serve as an effective therapeutic agent for treating tumors.

Natural compounds that are highly biologically active and have low toxicity are key players in the regulation of tumor metabolism (81). For instance, berberine, a natural compound, downregulates the expression of SLC1A5 in Hep3B and BEL-7404 cells, inhibiting Gln uptake and consequently suppressing expansion of liver cancer cells and tumor progression *in vivo* (77). Lobetyolin, a natural compound derived from Codonopsis, possesses anti-inflammatory and antioxidant properties (82). A recent study demonstrated that Lobetyolin disrupts Gln metabolism by downregulating SLC1A5 expression in MDA-MB-231 and MDA-MB-468 breast cancer cells, induces apoptosis in these cells and exerts anti-tumor effects (83).

In addition, numerous studies have demonstrated that several common clinical drugs can target SLC1A5 and inhibit tumor progression. Ivosidenib (Ag120) is a widely used clinical drug for advanced solid tumors and hematological malignancies. It targets tumor cells and inhibits tumor progression by reducing the production of 2-hydroxyglutarate (2-HG) mediated by isocitrate dehydrogenase 1 (IDH1mt) (84). Wei et al. found that Ag120 acts as an SLC1A5 inhibitor, blocking the uptake and metabolism of Gln, increasing autophagy and oxidative stress in colon cancer cells, and preventing the cancerous growth of CRC cells (85).

#### 4.2.2 Targeting Gln-related metabolic enzymes

In addition to Gln uptake inhibitors, enzymes involved in Gln metabolism also play a crucial role in tumor progression. Gln enters tumor cells through the action of transporter proteins and is converted into glutamate via dehydrogenation, catalyzed by GLS. Subsequently, glutamate enters the tricarboxylic acid cycle to generate energy. GLS is frequently overexpressed in various types of cancer, including TNBC, NSCLC, and acute myeloid leukemia (86–88), and is often linked to poor prognosis. Given the critical role of GLS in cancer metabolism and tumor progression, inhibiting GLS and targeting its breakdown have emerged as promising strategies for cancer treatment.

Therefore, inhibiting Gln degradation by downregulating the expression of these enzymes may represent a novel strategy for cancer treatment. GLS, one of the key enzymes in this pathway, is upregulated in various tumors and is thus considered a potential therapeutic target for cancer. Several GLS inhibitors have been developed, among which CB-839 is currently undergoing clinical trials as a potent, orally bioavailable GLS-selective inhibitor for the treatment of various cancers, including TNBC, NSCLC, and colon cancer (NCT03057600, NCT03057600, NCT03057600, NCT03057600, NCT03057600, NCT03057600). NCT02071862). In colon cancer, mutations in PIK3CA can increase the dependency of cancer cells on Gln. CB-839 preferentially inhibits the growth of colorectal tumors with PIK3CA mutations. Furthermore, the combination of CB-839 and 5-fluorouracil (5-FU) has been shown to induce regression in tumors with PIK3CA mutations. Mechanistically, CB-839 elevates the levels of intracellular ROS, enhances the movement of Nrf2 into the nucleus, leading to an increase in uridine phosphorylase 1 (UPP1) mRNA expression. UPP1 facilitates the conversion of 5-FU into its active compounds, thereby enhancing its inhibitory effect on thymidylate synthase. Additionally, data from a Phase I clinical trial (NCT02861300) indicated that the combination of CB-839 and capecitabine which is a prodrug of 5-FU, was well tolerated by CRC patients at bioactive doses. Moreover, CRC patients with PIK3CA mutations may experience more benefits from this treatment method compared to those with PIK3CA wild-type CRC (89).

As the consumption of Gln by cancer cells gradually increases, the Gln content in the TME (90). Consequently, tumor cells catalyze the synthesis of glutamate and ammonia into endogenous Gln by upregulating the expression of GS to sustain their growth and metastasis. Therefore, targeting GS may represent a viable strategy for combating cancer metastasis and improving survival rates in patients with malignant tumors. For instance, Long et al. discovered that in prostate cancer (PCa), vitamin C (VC) can induce oxidative stress and apoptosis by inhibiting GS. VC may serve as an effective anticancer therapy for cancers that are dependent on endogenous Gln (91).

### 4.3 Application of targeted Gln in combination with current antitumor therapies

Radiotherapy, chemotherapy, targeted therapy, and immunotherapy are the primary therapeutic modalities in the modern clinical treatment of malignant tumors, offering hope to many cancer patients. However, issues such as drug resistance and associated side effects have significantly restricted the clinical application of these treatments. Recent research has identified Gln metabolism as a promising target for enhancing the efficacy of malignant tumor therapies.

#### 4.3.1 Targeting Gln in combination with radiotherapy and chemotherapy

**Figure 3:**
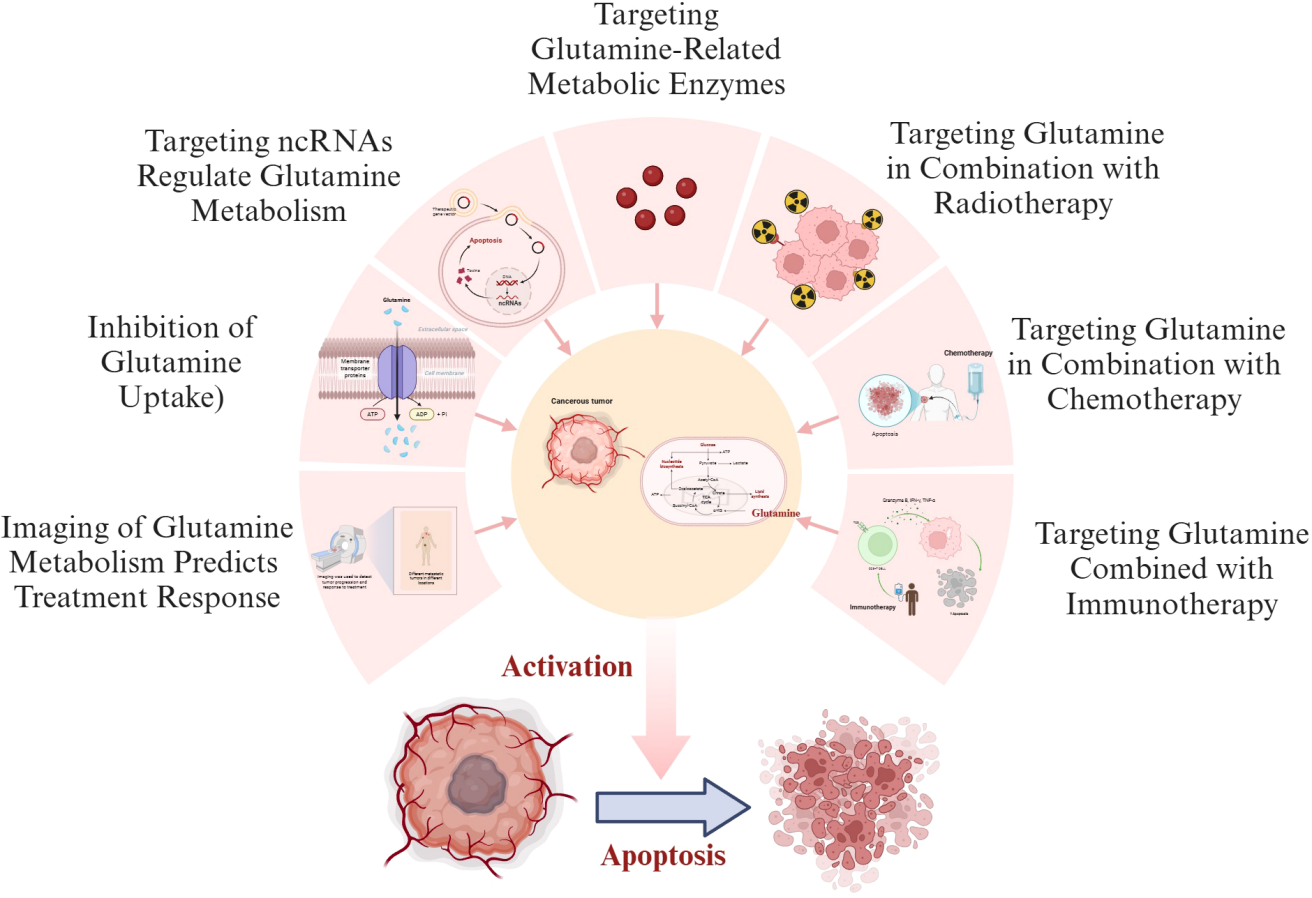
Gln targeted therapy combined with anti-tumor therapy. By inhibiting Gln transporters, ncRNAs, and metabolic enzymes, it exerts an anti-tumor effect by disrupting Gln metabolism. Targeted modulation of Gln metabolism can be effectively combined with radiotherapy, chemotherapy, and immunotherapy to enhance therapeutic synergy. Furthermore, abnormal Gln metabolism can serve as a predictive biomarker for prognostic responses to anti-tumor therapies, particularly when assessed using advanced imaging techniques.

Despite the remarkable clinical success of chemoradiotherapy and immunotherapy, many patients still do not benefit from these treatments. The modification of Gln metabolism is related to the ability of tumors to evade the immune system and reduced sensitivity to chemoradiation. Thus, blocking Gln metabolism could improve the effectiveness of various cancer treatments that target different pathways (**Figure 3**). In head and neck squamous cell carcinoma, Gln levels increase following radiotherapy, and the Gln transporter SLC1A5 is upregulated. Inhibition of Gln, when combined with radiotherapy, triggers ferroptosis in immunogenic tumors. The underlying mechanism involves radiotherapy increasing the expression of interferon regulatory factor (IRF) 1 by activating the interferon signaling pathway. IRF1 drives the expression of the transferrin receptor, promotes intracellular Fe2+ accumulation, disrupts iron homeostasis, and induces ferroptosis in cancer cells, while blocking Gln enhances this effect (92). Compared to mice with normal Gls1 expression, Gls1-deficient mice exhibited a significant increase in overall survival after radiotherapy, and their levels of natural killer cells and interferon alpha/gamma proteins were significantly higher than those of mice with normal Gls1 expression. This suggests that the mechanism of radiosensitization may involve the regulation of innate immunity through defective Gln metabolism (93). Mukha et al. discovered that radiation-resistant PCa cells and PCa stem cells (CSCs) have high Gln requirements (94). Enhanced Gln metabolism not only maintains the redox state of tumor cells and decreases their sensitivity to radiation but also sustains CSCs by regulating α-ketoglutaric acid (α-KG)-dependent chromatin-modifying dioxygenases. Inhibition of key regulators of Gln utilization, such as GLS and MYC, or reduced Gln consumption, has been shown to increase the radiosensitivity of PCa cells, diminish CSC formation *in vivo*, and decrease tumor development in xenograft mouse models. Importantly, the use of radiotherapy in conjunction with Gln metabolism inhibitors does not produce toxic effects on non-malignant prostate cells. Another study suggests that PCa cells resistant to Gln inhibition may also resist radiation-induced damage by activating the ATG-mediated autophagy pathway (95). Therefore, combining Gln metabolism inhibition and autophagy blockade with radiotherapy may represent a promising treatment strategy for PCa. Additionally, inhibiting Gln metabolism can reverse chemotherapy resistance. Kim et al. found that inhibiting Gln uptake increases the sensitivity of ovarian cancer cells to paclitaxel by blocking the activation of the mTORC1/S6K signaling pathway, downregulating Mcl-1 levels, and increasing Bcl-2 phosphorylation (96).

#### 4.3.2 Targeted Gln combined immunotherapy

In the tumor microenvironment, competition for Gln between tumor cells and immune cells significantly impacts anti-tumor immunity. Consequently, extensive research has focused on developing strategies that combine Gln targeting with immunotherapy to enhance the efficacy of immunotherapeutic approaches in tumors. Inhibiting Gln metabolism can impair SERCA activity by reducing intracellular GSH levels, which may lead to the increased expression of PD-L1.

Combined therapy that involves the inhibition of Gln metabolism alongside PD-L1 blockade has been shown to enhance the anti-tumor immune response of T cells (97, 98). Tang et al. discovered that molybdenum disulfide (MoS2), a highly stable and biocompatible nanomaterial, can effectively support anti-PD-L1 antibodies (aPDL1) and V9302, reduce Gln uptake by tumor cells, and increase the presence of activated CD8+ T cells within tumors. Furthermore, it promotes the migration of CD8+ T cells from the tumor periphery to the tumor core, thereby improving the efficacy of immunotherapy (97). In the context of gastric cancer, GPNA inhibits Gln uptake by targeting SLC1A5, thereby enhancing the anti-tumor effects of cetuximab both *in vivo* and *in vitro (* 99).

DON, which exhibits high structural similarity to Gln, enhances the cytotoxic effects of CD8+ T cells while inhibiting Gln metabolism in cancer cells. It has demonstrated significant anti-cancer efficacy in both preclinical and clinical trials. However, its clinical application has been limited by dose-dependent gastrointestinal toxicity observed in later phase I and II clinical trials. Given its potential, extensive research is being conducted to develop prodrugs of DON to improve cancer treatment outcomes. Among these, JHU395, a prodrug of DON, shows superior cell penetration compared to DON itself. At concentrations lower than those of DON, JHU395 has been found to decrease the growth and increase the apoptosis of high-MYC medulloblastoma cell lines, thereby prolonging the survival of treated animals (100). DRP-104 is a prodrug of DON that is currently undergoing clinical trials (NCT04471415). It is preferentially converted to DON within tumors while being inactivated as a non-toxic metabolite in gastrointestinal tissues (11). Metabolomic analyses have demonstrated that DRP-104 inhibits Gln metabolism in tumors and reduces the production of immunosuppressive metabolites. Furthermore, treatment with DRP-104 has been shown to increase immune cell infiltration and promote the polarization of TAMs into the M1 phenotype. Therefore, DRP-104, either as a monotherapy or in combination with immune checkpoint blockade therapy, may enhance clinical cure rates in cancer patients (101).

GLS1 is the rate-limiting enzyme responsible for the breakdown of Gln. It is regarded as a promising target for the treatment of tumors that are dependent on Gln (35,381,197). CB-839 is the most advanced GLS1 inhibitor currently in clinical practice and has been shown to effectively inhibit tumor proliferation (102). While CB-839 inhibited the proliferation of patient-derived glioma cell lines, it did not affect cell viability (103).In NSCLC, KEAP1 mutant NSCLC cell lines exhibit a dependence on Gln, and the GLS inhibitor CB-839 can inhibit Gln metabolism to exert anti-tumor effects (104). Beyond its direct impact, CB-839, when combined with immunotherapy, has been shown to inhibit the clonal expansion and activation of CD8+ T cells in NSCLC, thereby counteracting the adverse effects associated with PD-1 immunotherapy (12). In CRC, approximately 30% of cases involve PIK3CA mutations (38,194,275). These mutations increase the reliance of CRC on Gln metabolism. The combination of CB-839 and 5-FU resulted in significant regression of CRC in PIK3CA mutant xenografted nude mouse models (89). Furthermore, in addition to targeting Gln-dependent tumor cells, the combination of CB-839 and 5-FU enhances the secretion of IL-8 by cancer cells, which strongly induces the recruitment of neutrophils. These neutrophils form extracellular traps that actively kill cancer cells by inducing apoptosis (105). In patients with locally advanced, metastatic, and/or refractory solid tumors and hematological malignancies, CB839 is being evaluated in open-label phase I clinical trials (NCT02071862, NCT02071888, NCT02771626). These trials are currently ongoing and aim to address the issue of drug resistance while minimizing adverse reactions in clinical settings.

L-asparaginase can deplete plasma Gln and is widely utilized as an adjuvant therapy in lymphoma (106). Clinical studies have demonstrated its efficacy in patients with recurrent metastatic nasopharyngeal carcinoma. The use of L-asparaginase significantly enhances the effectiveness of immune checkpoint blockade therapy by improving CD8+ T cell adaptability. This combination represents a promising avenue for clinical application (107). *In vitro* experiments on lung cancer cells revealed that L-asparaginase treatment significantly induced autophagy activation in A549 cells, leading to their apoptosis (108). The application of L-asparaginase as a plasma Gln-depleting agent with a clear therapeutic effect in solid tumors warrants further investigation.

## 5 Discussion

Metabolic reprogramming is essential for the advancement of tumors and has become a central topic in cancer research lately (109). Cancer cells undergo metabolic reprogramming to sustain cell viability and growth in hypoxic and nutrient-deficient environments. A prominent example of this phenomenon is the Warburg effect, which describes how tumor cells preferentially utilize glucose for glycolysis even in the presence of sufficient oxygen, thereby supporting their rapid proliferation (110). In addition to glucose metabolic reprogramming, Gln metabolic reprogramming plays a crucial role in tumor metabolism, influencing tumor growth, metastasis, and drug sensitivity through various mechanisms. These mechanisms include the modulation of signaling pathway activation, DNA methylation, and the reconfiguration of the immune microenvironment. In tumor cells, oncogenes, tumor suppressor genes, and non-coding ncRNAs alter Gln metabolism by directly or indirectly regulating the expression and enzymatic activity of Gln-related genes. Consequently, the direct regulation of transporters and enzymes within metabolic pathways, as well as the indirect regulation of Gln metabolism through the modulation of associated oncogenes and ncRNAs, may represent promising avenues for future cancer therapies. Furthermore, the Gln metabolic status of cancer patients can be assessed using PET imaging and metabolomics analysis, helping to identify patients who may benefit from Gln metabolism suppression for more precise and individualized treatment.

While substantial progress has been accomplished in the study of tumor Gln metabolism in recent years, several key issues remain to be addressed (1). Cancer cells exhibit high heterogeneity, and various tumor types possess distinct metabolic mechanisms. For instance, in pancreatic ductal carcinoma (51), Gln deprivation promotes EMT progression and metastasis, whereas in ovarian cancer, Gln deprivation inhibits the cells’ ability to migrate and invade. Consequently, the potential differences in Gln metabolism among different tumor types require further clarification (2). Clinical trials have proven that consuming Gln by mouth can mitigate the incidence and severity of mucositis associated with radiotherapy and chemotherapy, thereby improving patient compliance with these treatments (111, 112). Additionally, Gln supplementation may enhance immune function and decrease the occurrence of complications following radical tumor surgery (113). It is essential to identify the role of Gln metabolism at various stages of cancer and evaluate its overall efficacy to achieve optimal clinical outcomes. (3) A substantial body of evidence indicates that the anti-tumor effect of targeting Gln metabolism alone is quite limited. In contrast, the combination of chemoradiotherapy and immunotherapy with Gln metabolism inhibition may yield a more pronounced anti-tumor effect. Therefore, various combination treatment options are being explored to maximize drug efficacy while minimizing side effects.

Although the clinical trial results for Gln metabolism inhibitors have not been satisfactory thus far, a substantial body of prior research has provided a scientific foundation for investigating the targeting of tumor Gln metabolism. This suggests that targeted therapy aimed at Gln metabolism may represent a novel and potentially effective strategy for cancer patients. Hence, the function of Gln metabolism in diverse cancer types, cell types, and individual cancer cases should be further explored to optimize the efficacy of Gln-targeted therapies in oncology.
